# Curcumin ameliorates gestational diabetes in mice partly through activating AMPK

**DOI:** 10.1080/13880209.2019.1594311

**Published:** 2019-04-07

**Authors:** Xuehong Lu, Fei Wu, Mengxue Jiang, Xiujuan Sun, Geng Tian

**Affiliations:** a Department of Kidney, The Second Hospital of Jilin University, Changchun, China;; b Department of Obstetrics and Gynecology, The Second Hospital of Jilin University, Changchun, China

**Keywords:** GSH, SOD, CAT, AMPK, HDAC4, Glucose-6-phosphatase

## Abstract

**Context:***In vitro* and *in vivo* research has shown that curcumin can alleviate diabetes and the relevant complications.

**Objective:** To investigate the effect of curcumin on gestational diabetes (GD).

**Materials and methods:** C57 BL/KsJ*^db^*^/+^(*db*/+) mice and C57 BL/KsJ^+/+^ mice (10–12 weeks old) were divided into four groups (*n* = 15): normal pregnancy (C57 BL/KsJ^+/+^), GD (C57 BL/KsJ*^db^*^/+^), GD plus low dose curcumin (50 mg/kg, orally gavage every day) and GD plus high dose curcumin (100 mg/kg, orally gavage every day). The tolerance of glucose and insulin were measured on gestation day 10. Body weight at birth and litter size of offspring were investigated, and the expression of oxidative stress factors [thiobarbituric acid reactive substance (TBARS), glutathione (GSH), superoxide dismutase (SOD) and catalase (CAT)] and AMP-activated protein kinase (AMPK), phospho-AMPK, histone deacetylases 4 (HDAC4), pHDAC4 and glucose-6-phosphatase (G6Pase) in the livers were analyzed by ELISA and Western blot on gestation day 20.

**Results:** High dose curcumin could partly ameliorate the intolerance of glucose and insulin, and completely restore the litter size and the body weight of GD mice through decreased TBARS expression (*p* < 0.05) and increased GSH, SOD and CAT expression (*p* < 0.05). Enhanced AMPK activation, accompanied with decreased HDAC4 and G6Pase expression (*p* < 0.05) were partly contributed to the alleviation of GD mediated by curcumin.

**Conclusions:** Although further detailed mechanism needs to be deciphered, curcumin can be considered as an alternative treatment for gestational diabetes.

## Introduction

Gestational diabetes (GD), the most common metabolic disorder during pregnancy, affects 2–5% of normal pregnancies (Hunt and Schuller 2007; Lipscombe and Hux 2007). GD can cause maternal morbidities, such as preeclampsia, polyhydramnios and operative deliveries (Sudharshana et al. 2018). The offsprings of GD are inclined to have adverse effects including prematurity, birth trauma, macrosomia, respiratory distress syndrome, and more importantly, will show a relatively high trend to develop dysregulated glucose tolerance. In the long term, the most serious hazard of GD is that the second generations will develop type 2 diabetes mellitus (T2DM) with a high tendency. GD is thus considered as a prediabetic state, providing the opportunity to study the abnormalities that may appear early in T2DM.

Due to its inhibition role against oxidative stress, inflammation and insulin resistance, curcumin has been demonstrated to alleviate diabetes and the relevant complications in many *in vitro* and *in vivo* research (Shao et al. 2012; Somlak et al. 2012; Castro et al. 2014; Green et al. 2014; Nabavi et al. 2015; Wang et al. 2016). In view of the similar aetiology and symptoms between diabetes and GD, curcumin, with its effects on diabetes, is highly likely to have similar physiological and pharmacological effects in alleviating GD. Little research has been done to verify whether curcumin could be used to treat GD.

In this study, we show that curcumin could ameliorate GD in mice, which may be through glucose metabolism by activating AMP-activated protein kinase (AMPK)/histone deacetylases 4 (HDAC4)/glucose-6-phosphatase (G6Pase) pathway.

## Methods and materials

### Chemical

Curcumin (Cur) ordered from Sigma-Aldrich (St. Louis, MO) was dissolved in dimethyl sulfoxide.

### Animals and study design

Six- to eight-week-old C57BL/KsJ^db/+^ (*db*/+) and C57BL/KsJ^+/+^ (wild-type) mice ordered from Jackson Laboratories were breed and fed with the chow diet (17% fat, 47% carbohydrate and 29% protein) (Harlan Teklad, South Easton, MA). All the protocols were sanctioned by the Ethics Committee of the Second Affiliated Hospital of Jilin University. Female mice (10–12 weeks) were individually mated and the next day was designated as gestation day (GD) 0. The gestated mice were grouped into four groups (15 mice in each group): normal pregnancy control (C57 BL/KsJ^+/+^, wild-type mice), GD group (C57 BL/KsJ*^db^*^/+^, *db*/+ mice), GD plus low dose curcumin group (50 mg/kg, orally gavage every day) and GD plus high dose curcumin group (100 mg/kg, orally gavage every day). 0.1% DMSO in saline was orally gavaged in the normal pregnancy control group and GD groups.

### Intraperitoneal glucose and insulin tolerance (IPGTT/IPITT) test

On gestation day 10, IPGTT and IPITT were performed. For IPGTT, glucose administration (2.0 g/kg, intraperitoneal injection, *i.p*.) was applied after 6 h fast. Then, blood glucose concentrations were analyzed using an ACCU-CHEK advantaged glucometer (Roche Diagnostics, Risch-Rotkreuz, Switzerland) and recorded at baseline and after glucose injection (30, 60, 90 and 120 min). For IPITT, insulin (0.75 U/kg) was intraperitoneally injected after 6 h fast and the glucose concentrations in the blood were measured at baseline and after insulin injection (30, 60, 90 and 120 min).

### Measurement of hepatic glycogen content

A commercial kit was utilized using the manufacture’s (BioVision, San Francisco, LA) protocols and expressed as mg/g protein. In brief, after combined with assay reagent, the absorbance of liver glycogen at 340 nm on a Spectra Max 384 spectrophotometer (Molecular Devices, Sunnyvale, CA) was measured and liver glycogen content was determined.

### Serum glucose, insulin and body weight measurement

Glucose level was analyzed with a glucometer (Roche Diagnostics) and plasma insulin was assayed with Ultra-Sensitive Mouse Insulin ELISA kit (ALP CO Diagnostics, Salem, NH) at gestation day 20. A top-loader balance (Thermo Fisher Scientific, Inc., Rockford, IL) was applied to calculate the body weight.

### Evaluation of oxidative stress

Catalase (CAT), thiobarbituric acid reactive substance (TBARS), glutathione (GSH) and superoxide dismutase (SOD) levels were determined with commercially available kits (Nanjing Jiancheng, Nanjing, China).

### Western blot

Rabbit anti-mouse primary antibodies specific for AMPK, phospho-AMPK, HDAC4, p-HDAC4, G6Pase, and α-tubulin (Santa Cruz, Dallas, TX) were used to incubate the respective proteins at 4 °C overnight after blocking. Then, the membrane was incubated with the peroxidase-conjugated secondary antibody (Sigma-Aldrich) at room temperature for 1 h and developed with an ECL system (GE Healthcare Life Sciences, Little Chalfont, Buckinghamshire, UK). NIH-Image J1.51p 22 (National Institutes of Health, Bethesda, MD) was applied to calculate the relative expression.

### Liver G6Pase activity

Microsomes were extracted from frozen-liver samples as previously described (Yamashita et al. 2003). Mannose-6-phosphate (1 mM) was utilized to assess the integrity of the microsomal membranes and G6Pase activity of microsomes was measured.

### Statistical analysis

All values in this study were shown as mean ± SEM and two-tailed Student’s *t*-test between different groups was performed; *p* < 0.05 was set as statistically significant.

## Results

### Curcumin relieves glucose and insulin intolerance in GD mice

**Figure 1.**
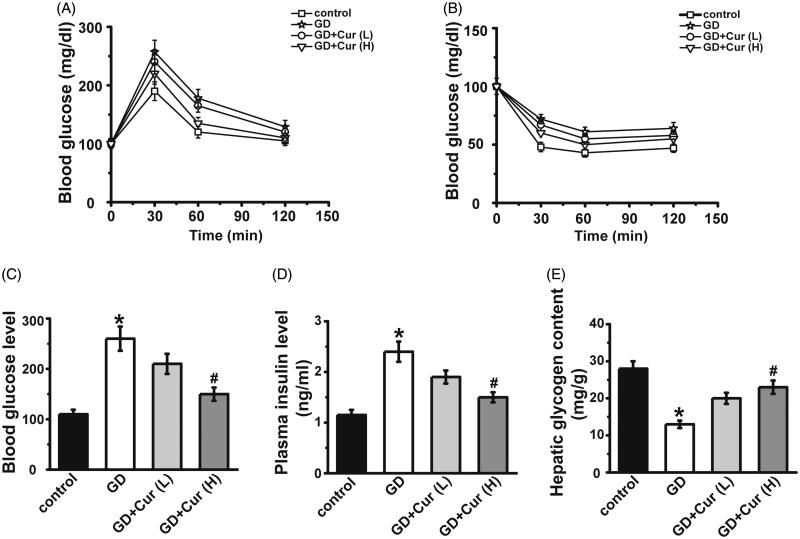
Effects of curcumin (Cur) on gestational diabetes (GD) mice. On gestation day 10, both glucose (A) and insulin (B) tolerance were measured. On gestation day 20, fasting blood glucose (C) and insulin (D) levels were detected and hepatic glycogen content (E) was measured. Cur: curcumin; GD: gestational diabetes; GD + cur (L): GD mice administrated with cur (50 mg/kg); GD + cur (H): GD mice administrated with cur (100 mg/kg). *p < 0.05, compared with control; #*p* < 0.05, compared with GD.

On gestation day 10, glucose and insulin tolerance were assayed by IPGTT and IPITT. The glucose level in the GD group was nearly 1.4-fold higher than that in the normal pregnancy control group (*p* < 0.05), while high dose curcumin treatment could partly inhibit the up-regulated blood glucose level (*p* < 0.05) (Figure 1(A)). Moreover, the glucose level in GD mice was notably higher than normal pregnancy control group at different time points (30, 60, 90 and 120 min) after insulin administration (Figure 1(B)), and as expected, the administration of high dose curcumin could significantly down-regulate the increase of glucose (Figure 1(B)). All these data indicated that curcumin ameliorated the intolerance of glucose and insulin.

On gestation day 20, both fasting blood glucose and insulin in GD mice were notably higher (about 2.5-fold) than that in the normal pregnancy control group (Figure 1(C,D)). The up-regulated fasting blood glucose and insulin levels in GD mice were significantly inhibited by high dose curcumin treatment (*p* < 0.05) (Figure 1(C,D)). Glycogen content in liver was remarkably down-regulated (nearly one half) in GD mice when compared with normal pregnancy control (Figure 1(E)), and high dose curcumin treatment could restore the decrease (*p* < 0.05) and low dose curcumin did not show a significant effect (*p* > 0.05).

### Curcumin attenuates oxidative stress in GD mice

**Figure 2.**
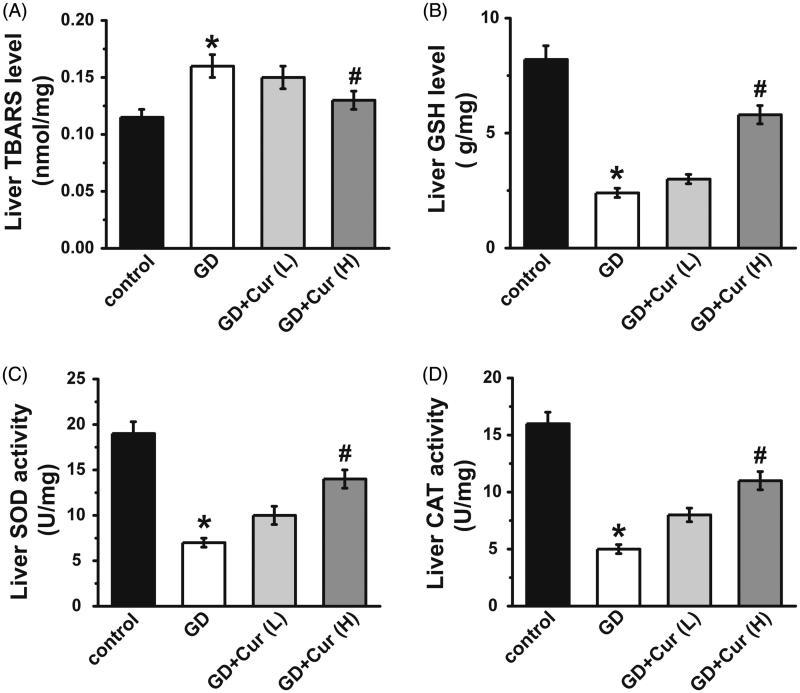
Curcumin (Cur) alleviates oxidative stress on gestational diabetes (GD) mice. TBARS (A), GSH (B), SOD (C) and CAT (D) activities were assayed. GSH: glutathione; TBARS: thiobarbituric acid reactive substance; CAT: catalase; SOD: superoxide dismutase. *p < 0.05, compared with control; #*p* < 0.05, compared with GD.

TBARS levels in livers of GD mice were significantly increased (nearly 1.3-fold) when compared with the normal pregnancy group (Figure 2(A)). The treatment of high dose curcumin significantly down-regulated the increased TBARS (80%, Figure 2(A)). GSH, SOD and CAT contents in GD mice were remarkably decreased (about 30%) in GD mice compared with normal pregnancy group (Figure 2(B–D)), while high dose curcumin markedly recovered such decrease (Figure 2(B–D)).

### Curcumin promotes liver AMPK activation in GD mice

**Figure 3.**
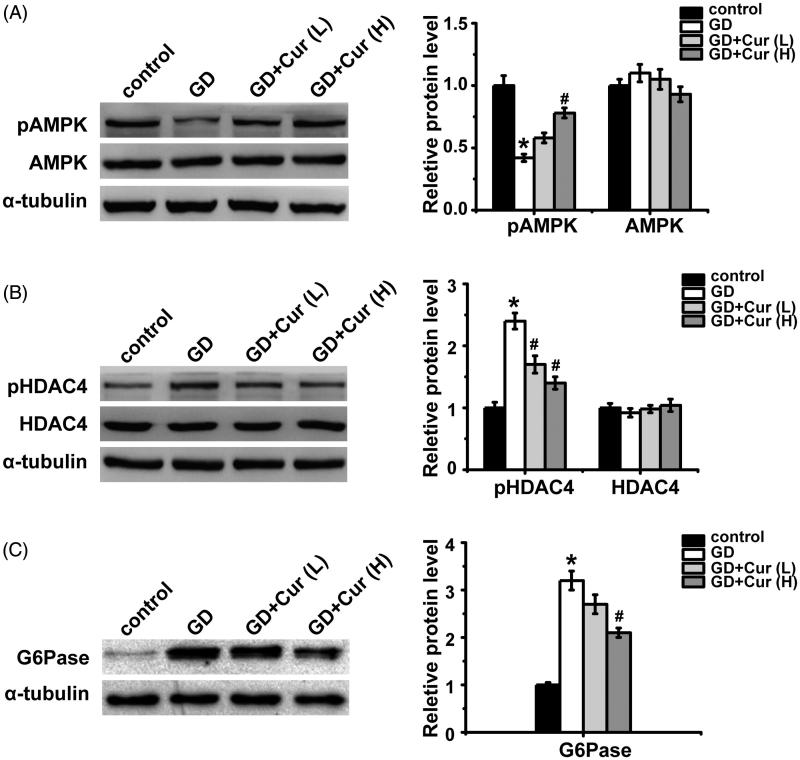
Effects of curcumin (Cur) on AMPK signaling pathway in gestational diabetes (GD) mice. The expression levels of total AMPK, p-AMPK (A), pHDAC4, total HDAC4 (B) and G6Pase (C) were detected by Western Blot in the livers of pregnant mice on gestation day 20. (p)AMPK: (phosphor-) AMP-activated protein kinase; (p)HDAC4: (phosphor-) histone deacetylases; G6Pase: glucose-6-phosphatase. *p < 0.05, compared with control; #*p* < 0.05, compared with GD.

Six mice from different groups at GD 20 were sacrificed and their livers were harvested. AMPK activation (Figure 3(A)) attenuated (nearly 60%) in GDM mice (*p* < 0.05), contributing to higher HDAC4 activation (about 2.5-fold) (Figure 3(B)) (*p* < 0.05), which could further up-regulate G6Pase expression (about 3.2-fold) (Figure 3(C)) (*p* < 0.05). High dose curcumin treatment could fully reverse AMPK related proteins activation pattern to reduce G6Pase expression (Figure 3). All of these indicated that curcumin could ameliorate GD in mice partly through activating AMPK.

### Curcumin improves the reproductive outcome of GD mice

**Figure 4.**
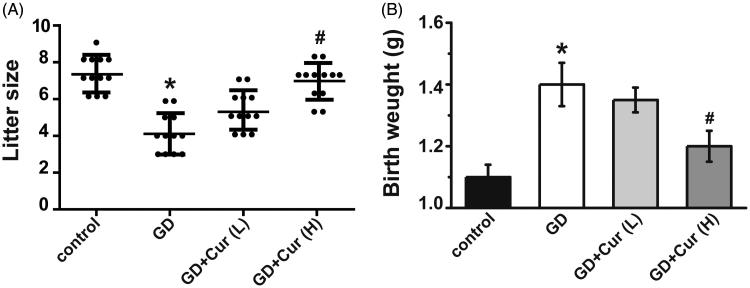
Curcumin (Cur) alleviates gestational diabetes (GD) reproductive outcome. Litter size (A) and body weight after birth (B) were recorded in different experimental groups (*n* = 12). *p < 0.05, compared with control; #*p* < 0.05, compared with GD.

High dose curcumin could completely restore the litter size decreased in the GD group (*p* < 0.05) (Figure 4(A)). Consistent with previous reports (Yamashita et al. 2001), GD could cause increased body weight (about 1.4-fold) when compared with wide type mice (*p* < 0.01) (Figure 4(B)), while high dose curcumin could decrease the body weight increased in the GDM group (*p* < 0.01) and restore the body weight to a normal level.

### Curcumin increases liver G6Pase activity in GD offspring

**Figure 5.**
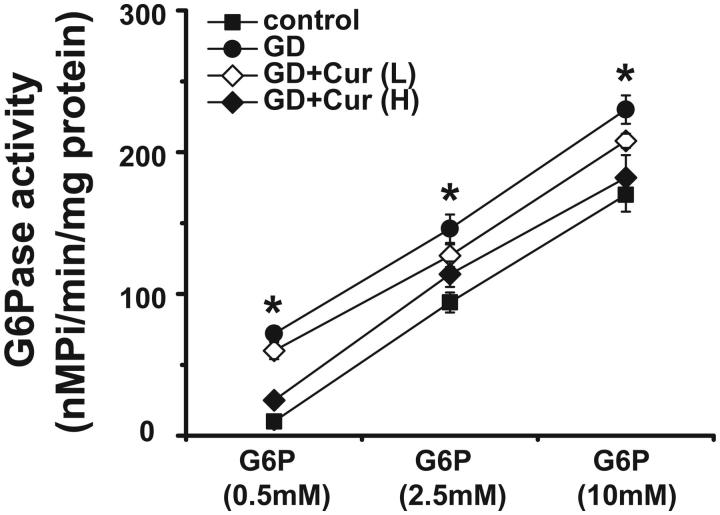
Effects of curcumin (Cur) on glucose-6-phosphatase activity in gestational diabetes (GD) offspring. Glucose-6-phosphatase activity in the liver of offspring was measured at birth (*n* = 12). *p < 0.05, compared with control and GD + cur groups.

Twelve offspring from different groups were chosen and livers G6Pase activity was assayed. High dose curcumin administration could markedly down-regulate the increased G6Pase activity in GD offspring (*p* < 0.05) (Figure 5). This result accompanied with previous data suggested that curcumin could partly activate AMPK pathway to reduce the expression and activity of G6Pase.

## Discussion

Besides insulin-mediated glucose regulation, the cell’s energy balance also influences glucose production (Biddinger and Kahn 2006). In this study, the promotion of reproductive outcome and the glucose and insulin tolerance mediated by curcumin are investigated in GD mice. Curcumin administration not only regulates oxidative stress (TBARS, SOD, GSH, and CAT) in the livers but also increases AMPK activation in the livers of GD mice. AMPK can act as an energy-sensing enzyme to switch metabolic phenotype from fat synthesis to fat oxidation, which will reduce the production of hepatic glucose and promote the uptake of muscle glucose. Consistent with previous reports of AMPK (Karpac and Jasper 2011; Mihaylova et al. 2011; O'Tierney-Ginn et al. 2013), our research also clarified that HDAC4 inhibition could be mediated by AMPK activation, which leads to down-regulation of G6Pase expression and activity. G6Pase is an enzyme which can hydrolyze glucose-6-phosphate into a phosphate group and free glucose.

Curcumin has been tested on aggressive non-obese diabetic models and streptozotocin-induced Type 1 diabetes models, which shows protection effects not only on *in vivo* islet damage but also on *in vitro* cultured β cells in the presence of proinflammatory cytokines (Kanitkar et al. 2008; Chanpoo et al. 2010; Castro et al. 2014). In addition to its powerful effects against oxidation, inflammation, and insulin resistance, high dose curcumin could prohibit glucose metabolism under normal culture condition in 3T3-L1 adipocytes (Ikonomov et al. 2002; Green et al. 2014; Zhang et al. 2016), whether such effect is also involved in physiological condition or in GD model needs further investigation. All of these researches indicate that curcumin may have multiple functions dependent on dose, targeted cell types, physiological and pathophysiological background. AMPK could be used as a pivotal biomarker for GD diagnosis and prognosis (Shao et al. 2012; Somlak et al. 2012; Castro et al. 2014; Weisberg et al. 2016). In this context, whether curcumin could directly target AMPK needs further investigated, which also warrants the research for other materials that can target AMPK to regulate glucose homeostasis and improve insulin sensitivity.

Reduced litter size and high body weight of offspring, as the two characteristic phenotypes in GD, are consistently observed in our study. It is noteworthy that the change in GD offspring body weight at birth is higher in mice than that in rats, which can be attributed by either species or facilities variations (Akyol et al. 2009). Our research clearly shows that curcumin could promote the fetus’ growth and development affected by GD.

Insulin is recommended to treat hyperglycaemia in the global guideline of pregnancy and diabetes. However, due to the unwelcome administration route and the potential to gain weight and hypoglycaemia, clinical practice is inconvenient (Yu 2017). Curcumin administration can not only relieve insulin resistance and hyperglycaemia in GD mice, but also significantly improve reproductive outcome and fetal development. Thus, curcumin might be an alternative treatment for GD.
